# Comparative Physiology of Menstruation

**Published:** 1884-01

**Authors:** Alfred Wiltshire

**Affiliations:** London


### THE COMPARATIVE PHYSIOLOGY OF MENSTRUATION.

BY ALFRED WILTSHIRE, M.D., F.R.C.P. LOND.

From the London Veterinary Journal.

*Continued from p.* 295, *Vol. IV.*

THE influence of domestication upon the fertility of animals amenable thereto is most instructive. The total productiveness of most of them is enormously increased thereby. The continuous and regular supply of food, the artificial warmth, care, and culture bestowed upon them, greatly enhance their productiveness. Puberty not only arrives earlier, but a sustained power of reproduction is acquired; more offspring can be bred, and these again breed earlier. The manifestation of "heat," therefore, instead of being confined to seasonal appearances, are much more frequently renewed, resembling, be it noted, those of women; and where celibacy is imposed upon the female, as it is for commercial purposes in the case of cows, for example, by segregation from the male, the periods of heat may be observed to recur with remarkable regularity–in cows, usually every three weeks. Domesticity, then, increases the fertility of domesticable creatures; while seclusion from the male, either for economic purposes or through captivity, permits the repeated exhibition in the female of the phenomena of the rut or heat, whatever they may be.

Prolonged civilisation has done for women what domestication has done for the lower animals; it has augmented their power of reproduction, made them more prolific, and rendered more frequent the manifestations of that aptitude, hence the sustained repititions of œstro-menstruation. Darwin (*Desent of Man*, p. 45) has remarked that savages appear to be less prolific than civilized people. Bischoff has truly said:—"If women menstruated only once or twice a year, it would long since have been remarked that such was the only time when conception was possible; menstruation would long ago have been recognized as perfectly analogous to 'the heat' in animals, even though the most essential element of it, the maturation of ova, had not been discovered." He also says: "The ova form and become mature, and are extruded from the maternal organism, usually at fixed periods, having regularly recurring intervals." This is the period of "heat" in animals and "menstruation" in human females. The analogies existing between menstruation in women and œstruation in the in the lower animals may be shown. In both there is special aptitude for conception at or near these times, though women are more highly endowed, inasmuch as they may conceive at epochs somewhat remote from their "period." The lower females will rarely permit intercourse except at periods of heat, which in ruminants, at any rate, are of brief duration; indeed, the males seldom attempt it during the intervals. This is unlike the practice of human beings. In both, it has been shown the germ-cells are then ready for impregnation; though here again women enjoys the advantage inplied above, in that ovules may be produced at times other than those of the "heat," that, however, being admittedly the usual and ordinary epoch for their ripening and dehiscence. Propinquity to the male, without access to him, is known to hasten ovulation in birds; and in the human female repeated sexual indulgence is known to stimulate the rupture, if no more, of Graafian follicles. One should not lose sight, however, of the superior evolution of the sexual system in the higher creatures, for this may not be without influence upon the process. It can hardly be maintained that sustained ovulation is the exclusive appanage of the higher mammals, but there can be little doubt that the organic superiority of the highest enables them to co-ordinate more readily the advantages their endowments and environments may chance to afford: their sexual superiority is correlated with their mental, as with their general organic superiority.

Bischoff says:—"In them (animals), the period of 'heat' either occurs so seldom, once or twice a year, or when repeated more frequently, as in the cow, the sheep, the pig, etc., it is so interfered with by ordinary circumstances, such as our economical arrangements and the like, that the animals are either immediately impregnated, or the recurrence of maturation of ova is obstructed and stopped by the secretion of milk. That analogy, otherwise so striking, with one of our commonest domestic animals, the cow, should have remained for the most part unnoticed, is no doubt to be attributed to some of the above circumstances."

To the question, should it be asked: Why do not the females of the lower animals menstruate with the persistent regularity with which the human female menstruates? the reply made be made that, under like advantages of food, warmth, and similar advantageous environments, they do so to a very large extent; but that, owing to their more constant exposure to conception, the catamenial function is liable to be suppressed by its natural successor, pergnancy. The regular periodical "horsing" of mares is often observed, especially when they are fairly well-fed and not over-worked; while cows, bitches, and other domesticated animals, exhibit regularly renewed signs of sexual aptitude. If it be asked: Do these signs resemble the sanguineous catamenial discharges of women? the reply is unequivocally affirmative; it is supported by reflection, and is confirmed by observation.

My researches have led me to certain conclusions, one of which, fundamentally important, may here be thus tentatively formulated. The sanguineous character of the œstro-menstrual flux bears generally some relation to the degree of evolution of the sexual system in mammalia. Ordinarily, it appears to be proportional with the development of the generative organs, and to be subordinate to their evolution; the chief evidence of evolution consisting in the lateral integration or fusion of the generative canals (Müller's ducts), accompanied by aggregation of the ovarian elements. But it should never be forgotten that intimately correlated therewith are other evolutionary facts, *e.g.*, the higher organization of the nervous, vascular, and glandular systems, upon the special activity or development of which variation may depend.

Accordingly, in the highest mammals, man and the quadrumana, wherein the uterus is a compact organ* (in the former. traces of the primitive duplicity being but feebly indicated), we find the catamenial flux to consist of almost pure blood. Even in the human species there are individual, often considerable, variations in the sanguineousness of the flux, in part obviously dependant upon the conditions of the blood—some women having only a feebly stained, or even a colourless flow, constituting the "menstruatio alba" of old authors.

In certain of the higher monkeys, in consonance with the law of evolutions already propounded, the catamenial flux is perfectly sanguineous, and recurs with regularity every three or four weeks. In cows it is also decidedly, though probably less intensely, sanguineous, and recurs with strict regularity every three weeks. Of the accuracy of this observation, as it applies to highly-bred cattle, I am assured by a most competent and able observer, Mr. Simpson, of Wray Park, Reigate, at whose model farm is a large herd of beautiful Alderney or Jersey cattle of his own breeding; and Mr. Simpson has not only for upwards of fifteen years been a breeder on a large scale (having a herd varying between 70 and 100 animals), but he brings cultivated powers of observation to bear upon the phenomena presented, having in his earlier years studied medicine.

More or less sanguineous (sanguineo-mucous) catamenial

---

* Gegenbaur says (*Elem. Comp.*, p. 616) :—"In the Simiæ, as in Man, there is a single uterus, received an oviduct on each side."

fluxes have also been observed to recur periodically in mares, asses, ewes, sows, bitches, cats and so on. Wild animals, kept in captivity, often sustain injury in their capacity for reproduction, but not always; and when they do manifest sexual aptitude, it has been observed to obey the law of periodicity (hebdomadal) already adduced. I am indebted to Mr. Bartlett, the able superintendent of the Zoölogical Gardens, for valuable information on this point. For instance, the hippopotamus, which has borne three young ones in the Gardens, exhibits monthly sexual excitement; the vulvar orifice swelling and weeping. Whether blood escapes is not certainly known, as the creatures frequent the water much at these times, copulation taking place in the water. Elephants, both male and female, become much excited during the breeding season; while some animals, at other times wild, become tame and quiet.

Observation shows that generally the discharge becomes less and less sanguineous as we go lower in the scale of organization, so that, in the lowest creatures, we see only a mucous exudation, perhaps but slightly, if at all, tinged with blood. In all presenting a vulvar aperture, there is swelling with turgescence of the genital orifice; but the exhalation of blood appears to be mostly, if not entirely, in relation with and subordination to the evolution of the sexual organs, so that, in the lowest forms, it may be represented mearly by an odorous mucous flux. Probably, undiscovered exceptions to this law exist. It is believed that their discovery would not invalidate the general truth of the law proponded; they would merely illustrate what is well known in biological inquiries, that variations and divergencies for adapted purposes are not uncommon in correlation with certain conditions of environment—nutritional, climatic, or other. Pouchet recognized some such relation as is indicated above, but he seems to have thought that it also bore relation to the size of mammals; and he regarded a copious intestini-form uterus, with its extensive mucous membrane, as accomodating a larger volume of blood than the thicker small uterus of woman and the Simiæ was capable of doing. He says: "The quantity of blood emitted by the animal is absolutely related to the structure of its uterus, but the afflux of the fluid towards the genital organ is always proportionate to the size of mammals. The species in which the genital apparatus more that of woman, and which as in monkeys, have a small and coriaceous uterus, are manifestly regular, and show an abundant external flow of blood. We have seen that the assertions of Buffon, Cuvier, Burdach, G. Sainte-Hilaire, Rengger, Ehrenberg, Raciborski, and Isidore Sainte-Hilaire, leave no doubt in this respect;" and again, "Thus, then, menstruation in the sow is a demonstrated fact. As in human species, there is an emition of blood; but if this is not abundant, it is because this fluid is found in great part poured in the immense extent of the internal generative apparatus."

The generalisation I have independently worked out, and now venture to propound, differs from Pouchet's in that I regard the relation of the catamenial function, in respect to its sanguineousness, to the generative organs as being, in the main, one of subordination to the extent of their evolution, not merely to the accidents of shape of the uterus or size of the creature. Aberrations in, exceptions to, or divergencies from the law, may in time be discovered, or may declare themselves in the course of inquiry; and the explanation of exceptions, apparent or real, may be found in, or be ascribable to, such causes as difference in the plasticity or adaptability of animals to their enviroment; in the tendencies of some species to organic degradation, or to other factors as yet unknown; but with the allowances scientific caution would prescribe, it is believed that the general accuracy of the evolutionary law, now provisionally advanced, will become established as observations are extended. Seeing the extreme difficulty of instituting adequate personal inquiry into the phenomena as they are presented in all animals by one individual, it is to be hoped that all who enjoy exceptional opportunities for observation, will be so good as to make and record them, so that in process of time, an accurate record of facts may be garnered.

With ample material furnishing reliable data, further conclusions may become warrantable, and correlations be discovered that are now unsuspected. For example, the flux may be found to bear some relation to the evolutionary or hæmoglobin value of the red cells. Probably, diet influences it also; and, possibly, it may bear some relation to the number of ova, for in the multiparous carnivora the manifestations of heat, including the flux, are more sustained than in the uniparous ruminant.

In illustration of my position, the following passages, borrowed from Gegenbaur, may be usefully quoted:

"Two completely separated uteri open into a vagina in many Rodentia (Lepus, Sciurus, Hydrochœrus, etc.,) and in Orycteropus. In other Rodentia, the two uteri are only united for a short distance into a common opening into the vagina (*e.g.*, Cavia, Cœlogenys, Mus). This leads to the arrangements seen in the uterus of the Insectivora, Carnivora, Cetacea, and Ungulata, where a single uterus is continued into two separate cornua, which are continued into the oviducts. When the common portion of the uterus is elongated, the cornua are shortened; this is the case in the Chiroptera and Prosimiæ; in the Simiæ, as in Man, there is a single uterus which receives an oviduct on either side. The cornua of the uterus, and the common uterus itself, vary greatly in length; so, too, does the vagina, the mucous membrane of which may be variously modified. In many Rodents (Logotomus), a certain portion retains its original double nature. Its opening into the urogenital sinus is sometimes distinguished by a temporary fold of mucous membrane, which is known as the hymen. This has been observed in the Ruminantia, Carnivora, etc.; but it is in the Simiæ only that it has the same relations as in man. The primitive Müllerian duct, which only served for the passage of the generative products, is therefor, differentiated into three parts, owing to the great physiological changes that happen to it; and of these parts, the first, or Fallopian tube, alone retains its primitive relations.

"The ovaries, which are generally small, vary greatly according to the relation that obtains between the follicles and the struma of the ovary. In a large number of mammals they are racemose in form. They seldom retain their primative position, and generally travel towards the pelvic basin, or, with their oviducts, or, rather, to its infundibular cœlomic mouth, for a process of the margin of the ostium extends to the ovary. The mesenteric folds (ligamenta uteri lata), which support the ovaries and overducts, and unfrequently unite with the pouch that encloses the ovary to form the mouth of the oviduct (as in the carnivora)."

We may now examine the evidence furnished by observation upon the females of the lower animals at their periods of heat. It will be apparent that there is singular accord in the statements of competent observers upon the phenomena as they present themselves in the various animals; and that, in all the creatures subjected to inquiry, some more or less conspicuous flux or exudation occurs; the majority exhibiting manifestations partaking in varying degrees of a sanguineous character.

In observing domesticated animals, and still more those in a state of captivity, the same allowances for individual variations should be made as would be made in regard of women; in whom we find laborious life and hard fare to some extent diminish the flow; while ease, luxury, and plenty (not excess) promote it, Again, racial peculiarities, with the lower animals as with the human species, may unquestionably affect the character of the flow.

Very slight changes in normal conditions affect the capacity for reproduction in all animals; hence, many creatures fail to breed in captivity, while, on the other hand, domesticable creatures have augmented powers. Mr. Darwin's statements to this effect are numerous and weighty. In "Animals and Plants under Domestication," vol. ii., pp. 143-4, he says:—"It would appear that any change in the habits of life, whatever these habits may be, if great enough, tends to affect in an inexplicable manner the powers of reproduction. The result depends more on the constitution of the species than on the nature of the change; for certain whole groups are affected more than others; but exceptions always occur, for some species in the most fertile groups refuse to breed, and some in the most sterile groups breed freely. . . . . Changed conditions of life have an especial power of acting injuriously on the reproductive system. The whole case is quite peculiar, for these organs, though not diseased, are thus rendered incapable of performing their proper functions, or perform them imperfectly." *Ibid.*, p. 256:—"We know that certain groups of organic beings, but with exceptions in each group, have their reproductive systems much more easily affected by changed conditions than other groups—for instance, carnivorous birds more readily than carnivorous mammals, and parrots more readily than pigeons; and this fact harmonises with the apparently capricious manner and degree in which various groups of animals and plants vary under domestication."

(*To be continued.*)

